# Epidemiological analysis and rapid detection by one-step multiplex PCR assay of *Haemophilus influenzae* in children with respiratory tract infections in Zhejiang Province, China

**DOI:** 10.1186/s12879-018-3295-2

**Published:** 2018-08-22

**Authors:** Xingli Fan, Xiaoxiang Liu, Lei Ji, Damin Cai, Jinqin Jiang, Jingjing Zhu, Aihua Sun, Jie Yan

**Affiliations:** 1 Department of Medical Microbiology and Parasitology, Zhejiang University School of Medicine, Hangzhou, 310031 Zhejiang China; 2 Department of Basic Medicine, Hangzhou Medical College, Hangzhou, 310058 Zhejiang China

**Keywords:** *Haemophilus influenzae*, Respiratory tract infections, Epidemiological analysis, Multiplex PCR, Nontypeable *H. influenzae*, *Encapsulated H. Influenzae*

## Abstract

**Background:**

*Haemophilus influenzae (H. influenzae)* is one of the most important pathogenic bacteria causing respiratory tract infection diseases in children. There are two main types of *H. influenzae*, encapsulated *H. influenzae* and nontypeable *H. influenzae* (NTHi). Serotype b of *H. influenzae* (Hib) used to be the main epidemic type of *H. influenzae*, causing the invasive infection. However, the epidemiology of invasive *H. influenzae* disease has changed substantially, and most invasive diseases are now caused by NTHi and other serotypes of *H. influenzae*. The aim of this study was to determine the main epidemic strains of *H. influenzae* in Zhejiang Province in China, and establish a one-step multiplex PCR assay to distinguish *H. influenzae* from other bacteria associated with respiratory tract infections, and distinguish encapsulated *H. influenzae* from NTHi.

**Method:**

In this study, bacterial culture and serum agglutination testing were used to determine the most prevalent serotype of *H. influenzae*, and the results have served as a gold standard for clinical diagnosis. We also designed a one-step multiplex PCR assay using several kinds of standard strains of respiratory tract infection bacteria, to examine the stability, specificity, and detection limit of the PCR assays. We then used 1514 nasopharyngeal secretion (NPS) samples collected from children with respiratory tract infections to verify the specificity and sensitivity of the PCR assay.

**Results:**

The bacterial culture and serum agglutination test results showed that the positive rates of *H. influenzae* and encapsulated *H. influenzae* were 18.49 and 1.18%, respectively. The PCR results showed that the detection limit of the multiplex PCR assay was 1.89 × 10^3^ copies /μL, the *ompP6* positive rate was 19.35%, and the *bexA* positive rate was 1.32%. The sensitivity and specificity of the multiplex PCR were 100 and 99.86%, respectively.

**Conclusions:**

According to our study, the most prevalent *H. influenzae* subtype in Zhejiang Province was NTHi, account for 93.57%; the one-step multiplex PCR assay we established can be used as the differential detection of clinical *H. influenzae* strains, replacing routine bacterial culture and serum agglutination testing.

## Background

*Haemophilus influenzae* (*H. influenzae*) is a Gram-negative coccobacillus that can cause invasive and severe infections, such as bacteria pneumonia, epiglottitis, sinusitis, and similar conditions, in both adults and children, especially in children under 5-years-old [1, 2]. There are two main types of *H. influenzae* based on the capsule: encapsulated *H. influenzae* and nontypeable *H. influenzae* (NTHi). The NTHi cannot be typed by the serum agglutination test. Serotype b *H. influenzae* (Hib) used to be the main type of *H. influenzae* in respiratory tract infections, but the epidemiology of invasive *H. influenzae* disease has changed substantially, with most invasive diseases now caused by non-b serotypes and NTHi strains [3–8]. Different types of *H. influenzae* have different pathogeneses and require different treatments [9, 10]. Detection of *H. influenzae* by traditional methods like bacterial culture is cheap and facilitates the detection of antibiotic resistance. However, it is time-consuming and may give false-negative results during ongoing antibiotic treatment. Serum agglutination testing was widely used to determine the serotype of encapsulated *H. influenzae*, and it can also be used to discriminate the encapsulated *H. influenzae* from NTHi. However, sometimes there are bacterial self-coagulation phenomena and cross agglutination, and serological reagents are more expensive.

Because the specificity of PCR depends on the selection of target genes, it is challenging to design a specific PCR test for the detection of *H. influenzae* and discriminate encapsulated *H. influenzae* from NTHi. The gene *ompP6*, which encodes the outer membrane protein P6, is present in all the *H. influenzae* strains and has a highly conserved nucleotide sequence. The nucleotide sequence homology of *ompP6* was 96.7–100%. *OmpP6* is a good target gene to distinguish *H. influenzae* from other respiratory tract infection bacteria [11–13]. The gene *bexA* encoding the capsule transport protein exists only in encapsulated *H. influenzae*, and the nucleotide sequence at site 3552–3865 is highly conserved [14–17]. According to the distribution and sequence of *ompP6* and *bexA*, we designed two pairs of primers to establish a one-step multiplex PCR assay for differential detection of clinical *H. influenzae* species from nasopharyngeal secretion (NPS) in patients with respiratory tract infections in order to improve the detection rate and identify the predominant *H. influenzae* strain in Zhejiang Province in China.

## Methods

### Subjects

This work has obtained the approval from the Research Ethics Committee of Hangzhou Medical College. The approval numbers are 2012–001.The nasopharyngeal secretion (NPS) used in this study have been allowed to collect from patients with respiratory tract infections. Written informed consents have been obtained from the participants.

One thousand five hundred fourteen NPSs were gathered from patients diagnosed with respiratory tract infection disease and treated in the Yiwu Women and Children’s Hospital and the Shaoxing City Keqiao District Hospital of Traditional Chinese Medicine from 2012 to 2016. They all diagnosed with respiratory tract infection, including 289 sinusitis, 108 bronchitis, 83 bronchopneumonia, 262 tonsilitis, 53 HFMD, 133 herpangina, 80 stomatitis, 80 submaxillary lymphadenitis and 426 other respiratory tract diseases. The patients ranged from 0 to 14 years old, and included 825 boys and 689 girls. One thousand fifty-five of them were 5 or under 5 years old, 459 of them were 6 or elder than 6. No antibiotics were used in the 72 h preceding admission. Under nasal endoscopy, nasal meatus secretions were obtained by cotton swabs from these patients, placed in sterile tubes, and immediately sent to the laboratory for pathogen isolation. The same amount of pH 7.6 1% trypsin was added to the samples and incubated for 90 min at 37 °C to homogenize the NPSs, and 200 μL of the homogenized sample was used for bacterial culture. The remains were kept at − 80 °C.

### Bacterial culture and identification

Here, 100 μL of each homogenized sample was inoculated on blood plates and chocolate plates under sterile conditions by zoning streak and incubated in 5% CO_2_ incubators at 37 °C for 24–48 h. The *H. influenzae* was screened preliminarily by colony morphology of and the characteristics of Gram staining, and then identified by V factor, X factor and V + X factors requirement test. NTHi ATCC49247, encapsulated *H. influenzae* standard strain ATCC 9327 (Type a), ATCC 9334 (Type b), ATCC 9007 (Type c), ATCC 9332 (Type d), ATCC 8142 (Type e) and ATCC 9833 (Type f), *Streptococcus pneumoniae* ATCC 49619, *Staphylococcus aureus* ATCC 29213, *Klebsiella pneumoniae* ATCC 700603, *Pseudomonas aeruginosa* ATCC 27853, *E. coli* ATCC 25922 were donated by department of pathogenic biology, Zhejiang University. The standard *H. influenzae* strains were cultured with selective medium and other standard strains were cultured on blood plates, in 5% CO_2_ incubators at 37 °C for 24–48 h.

### Serum agglutination test

The antisera of serotypes a, b, c, d, e, and f of *H. influenzae* were used to determine the serotype of *H. influenzae*, which we cultured from clinical samples using a slide agglutination test. Serotyping reagents were purchased from TBC in the United States (batch number: IM-H1015).

### DNA extraction

The fresh cultures of each standard strain were centrifuged after saline washing, and genomic DNA was extracted by using a Bacterial Genomic DNA Extraction Kit (GENEray). DNA from homogenized samples was also extracted using the same kit. The concentration and purity of DNA were determined by UV spectrophotometry.

### One-step multiple PCR assay design

In this PCR assay, we designed two sets of primers of *ompP6* and *bexA* according to the nucleotide sequence of *H. influenzae* type b reported on GenBank (Table 1). The expected *ompP6* amplified product was the whole 459 bp sequence of *ompP6* and the expected *bexA* amplified product was about 343 bp, only the conserved region at 3552–3895 site of *bexA* gene. All primers were purchased from Thermo Fisher Scientific, Inc. (China). The DNAs of NTHi standard strain ATCC49247, encapsulated *H. influenzae* standard strain ATCC 9327 (Type a), ATCC 9334 (Type b), ATCC 9007 (Type c), ATCC 9332 (Type d), ATCC 8142 (Type e), and ATCC 9833 (Type f), *Streptococcus pneumoniae* ATCC 49619, *Staphylococcus aureus* ATCC 29213, *Klebsiella pneumoniae* ATCC 700603, *Pseudomonas aeruginosa* ATCC 27853, and *E. coli* ATCC 25922 were used to develop the PCR assay. The PCR amplification was performed in 50 μL reactions containing: 2.5 mol/L of dNTP, 250 mol/L of each primer, 2.5 U of Ex-Taq DNA polymerase (Takara), 2 μL target DNA and 1 × PCR buffer. The PCR cycler was set to the following program: 4 min of enzyme activation at 94 °C, followed 35 cycles at 94 °C for 30s, and 54 °C for 30s and 72 °C for 45 s, followed by 1 cycle at 72 °C for 5 in. The PCR product was examined by 2% agarose electrophoresis.

**Table 1:** One-step multiple PCR primers

Oligonucleotide	Sequence
*ompP6* forward	5′-ATGAACAAATTTGTTAAATCA-3′
*ompP6* reverse	5′-TGCGATGTTGTATTCAGGTGTA-3′
*bexA* forward	5′-CGTTTGTATGATGTTGATCCA-3′
*bexA* reverse	5′-TGTCCATGTCTTCAAAATG-3′

### Repeatability and specificity of the PCR assay

The PCR were repeated three times under the same conditions. The PCR amplification of *ompP6* and *bexA* of standard strains were purified using a PCR product purification kit and then sequenced by Thermo Fisher Scientific, Inc. All the sequenced nucleotide sequences were compared using the sequence reported on GenBank.

### Detection limit of PCR assay

*H. influenzae* standard strain ATCC 9334 (type b) was used to determine the detection limit of the *ompP6* PCR assay and multiplex PCR assay. The DNA concentration of ATCC 9334 was diluted 10 times and added to the PCR reaction. The DNA concentration was 1.89 × 10^6^ copies/μL, 1.89 × 10^5^ copies /μL, 1.89 × 10^4^ copies /μL, 1.89 × 10^3^ copies /μL, and 1.89 × 10^2^ copies /μL, respectively. The lowest DNA concentration at which positive products could be amplified was considered the detection limit.

### Determination of *H. influenzae* in clinical samples by the multiplex PCR

We used the multiplex PCR assays we established to detect the *H. influenzae* subtype of the 1514 clinical NPSs.

### Statistical analysis

Statistical analysis was performed using IBM SPSS Statistics ver. 11.0 (IBM Co., Armonk, NY, USA). Statistical significance was defined as *P* < 0.05. Chi-square test was used in this study. The sensitivity and specificity of the multiplex were measured by comparison to the results of bacterial culture and serum agglutination.

## Results

### Bacterial culture results of 1514 NPSs from children with respiratory tract infections

Of 1514 NPSs from children with respiratory tract infection, 280 were positive for *H. influenzae* as indicated by bacteria culture, the positive ratio was 18.49%. The *H. influenzae* infection rates of boys and girls with respiratory tract infectious diseases were 20% (165/825) and 16.69% (115/689), and there was no significant difference in infection rate between boys and girls (χ2 = 1.2, *P* > .05) (Table 2).

**Table 2:** Results of bacterial culture and serum agglutination test

Bacterial culture	*H. influenza*-positive					*H. influenza*-negative	Total cases
Serotype	NTHi	Serotype a	Serotype b	Serotype c	Serotype f	1234	1514
	262	4	1	4	9		
Total cases	280						

### Results of serum agglutination test of *H. influenzae*

According to the serum agglutination test, there were 262 NTHi strains among all 280 *H. influenzae* strains cultured from NPSs, accounting for 93.57%. There were 18 strains of encapsulated *H. influenzae*, including 4 type a, 1 type b, 4 type c and 9 type f (Table 2).

### Specificity, stability, and detection limit of the PCR assay

All the *H. influenzae* standard strains had amplification of *ompP6* and the standard strains of *Staphylococcus aureus*, *Streptococcus pneumoniae*, *Klebsiella pneumoniae*, *E.coli*, and *Pseudomonas aeruginosa* showed negative results in the multiplex PCR assays. The encapsulated *H. influenzae* determined by serological test had amplification products of *bexA*. The PCR was performed in triplicate at different times and the same results were found. The results obtained here matched the *ompP6* and *bexA* nucleotide sequences reported on the GenBank. The detection limit of the multiplex PCR assay we used was 1.89 × 10^3^ copies /μL.

### *H. influenzae* detection in nasopharyngeal secretions (NPSs) with one-step multiple PCR assay

Two hundred seventy-three of 1514 NPSs were found to be positive only *ompP6* positive and 20 of 1514 were found to be positive for both *ompP6* and *bexA* when assessed using the multiplex PCR scenario established above. The sensitivity and specificity of the multiplex PCR were 100 and 99.86%, respectively (Table 3).

**Table 3:** Comparison of one-step multiple PCR and bacterial culture plus serum agglutination test of 1514 NPS

Item	bacterial culture + serum agglutination test			Specificity (%)	Sensitivity (%)
	*H. influenzae-*positive		*H. influenzae-*negative		
	NTHi	Encapsulated *H. influenzae*			
*ompP6*+, *bexA* +	2	18	0	99.86	100
*ompP6*+, *bexA* -	260	0	13		
*ompP6*-, *bexA* -	0	0	1221		
Total	1514				

## Discussion

*H. influenzae* is a common pathogen, causing acute respiratory tract infection and meningitis in children in most developing countries [18]. The epidemiology of invasive *H. influenzae* disease has changed substantially, with most invasive diseases now caused by non-b serotypes and NTHi strains [3–8].The incidence of infectious disease caused by Hib declined and that caused by other serotypes of *H. influenzae* and NTHi has increased over the past 10 years [4–7], especially in Europe and North America. NTHi caused 78% of all cases and showed increasing trends among persons < 1 month and > 20 years of age in Europe. Establishing the contribution of NTHi to respiratory tract infections may be helpful in the development of a new vaccine against NTHi that outweighs the cost of long-term healthcare in terms of repeated episodes and sequelae.

Drug resistance of bacteria is a serious problem.β-lactamase production in *H. influenzae* and NTHi is still the dominant resistance mechanism against β-lactams. The prevalence of β-lactamase in NTHi differs widely worldwide, strains with β-lactamase independent resistance to β-lactams have emerged and are spreading. The emergence and spread of β-lactamase-negative ampicillin-resistant strains in many regions of the world is of substantial concern, potentially necessitating changes to antibiotic treatment guidelines for community-acquired infections of the upper and lower respiratory tract and potentially increasing morbidity associated with invasive NTHi infections [19].

In recent years, the predominant strains of *H. influenzae* have varied across different regions of China, and the distribution of dominant strains in one place has also been affected by disease type, patient age, and similar factors. It is very important to quickly and accurately distinguish *H. influenzae* from other bacteria, or encapsulated *H. influenzae* from NTHi for timely treatment.

Zhejiang is a major economic province in China, located in southeastern China with a large population. In this study, we gathered 1514 NPSs and used traditional detection methods to determine the *H. influenzae* infection ratio and serotype of *H. influenzae* in Zhejiang Province in China. According to results of the bacterial culture and serum agglutination test, the *H. influenzae* infection rate in respiratory tract infections in children ranged from 0 to 14 years was 18.48% (280/1514), of all the *H. influenzae* we detected from clinical samples, 93.57% (262/280) belonged to NTHi, only 6.43% (18/2280) were encapsulated *H. influenzae*, and only 0.36% were Hib. It means NTHi is the predominant strain of *H. influenza* in children respiratory tract infection in Zhejiang province, China.

Because *H. influenzae* is a fastidious bacterium, the rate of detection of *H. influenzae* with routine bacterial culture is low. Serum agglutination tests have become widely used to determine the serotype of encapsulated *H. influenzae*. But sometimes there are bacterial self-coagulation phenomena and cross agglutination, and serological reagents are more expensive. In recent years, PCR has become widely used to detect pathogenic microorganisms in different samples, especially bacteria which are difficult to culture under routine conditions.

Considering of these, we also established a one-step multiple PCR assay by detecting the ompP6 and bexA genes, and we used several kinds of standard strains of respiratory tract infection bacteria, including H. influenza, to examine the stability, specificity, and detection limit of the PCR assay. The results showed that the PCR assay had high stability and specificity, and the detection limit of the PCR assay was 1.89 × 103 copies /μL. The 1514NPSs were used to verify the specificity and sensitivity of the PCR assay. In this study, 293 of 1514 NPSs were ompP6- positive, the detection rate of H. influenzae was 19.35% (293/1514). The *H. influenzae* positive rate of one-step multiple PCR is higher than bacterial culture, it’s the reason why we want detect *H. influenzae* by PCR instead of bacterial culture because *H. influenzae* is a fastidious bacterium and maybe there are some false negative.

Twenty of 1514 NPSs were bexA gene-positive, and the positive rate was 1.32%, compared with the serum agglutination test, 2 NTHi were missed diagnosed. It may be a new finding, and it may be the key note for further researches in PCR and molecular characterization.

Compared with the results of bacterial culture and the serum agglutination test, the sensitivities of *ompP6* and *bexA* detection were both 100%, and the specificity of *the* multiplex PCR was 99.86%. There was no significant difference in the rate of detection of *H. influenzae* between the multiplex PCR and the routine bacterial culture (18.49%).

According to our study, and considering the shortages of bacterial culture and serum agglutination test, the one-step multiplex PCR assay we established can be used to identify encapsulated *H. influenzae* and NTHi in place of routine bacterial culture and serum agglutination testing, but it cannot be used to determine the serotype of encapsulated *H. influenzae*. It is necessary to design new, effective, specific typing primers, which will require further research.

## Conclusions

According to our study, the infection rate of H. influenzae in respiratory tract infections was 18.49%; the most prevalent subtype of *H. influenzae* in Zhejiang Province was NTHi, account for 93.57%; the sensitivity and specificity of the one-step multiplex PCR assay we established were 100 and 99.86%, respectively; the multiplex PCR can be used as the differential detection of clinical *H. influenzae* strains, replacing the routine bacterial culture and serum agglutination testing.

## Abbreviations

H. influenzae: Haemophilus influenzae
HFMD: hand foot mouth disease.
NPS: nasopharyngeal secretion
NTHi: nontypeable *Haemophilus influenzae*
